# Prospective Multicenter Propensity Score-matched Comparison of Ultrasound-guided Versus Endoscopic Carpal Tunnel Release

**DOI:** 10.1016/j.jhsg.2026.100974

**Published:** 2026-02-27

**Authors:** Victor M. Marwin, Johnny T. Nelson, James F. Watt, James R. Verheyden, Paul E. Perry, Lance G. Warhold, David J. Carl, M.J. Palmer, Steven R. Niedermeier, Larry E. Miller, Jenna M. Godfrey

**Affiliations:** ∗ Bluegrass Orthopaedics, Lexington, KY; † The Bone & Joint Surgery Clinic, Raleigh, NC; ‡ Orthopaedic Associates, Fort Walton Beach, FL; § Center for Orthopedics & Neurosurgery, Bend, OR; ‖ Tri-State Orthopaedics, Evansville, IN; ¶ Dartmouth Hitchcock Medical Center, Lebanon, NH; # Meadville Medical Center, Meadville, PA; ∗∗ Prisma Health-Upstate, Greenville, SC; †† TMI Sports Medicine & Orthopedic Surgery, Arlington, TX; ‡‡ Miller Scientific, Johnson City, TN; §§ Slocum Research & Education Foundation, Eugene, OR

**Keywords:** Carpal tunnel release, ECTR, Endoscopic, UGCTR, Ultrasound

## Abstract

**Purpose:**

To compare 3-month clinical outcomes of patients treated with ultrasound-guided carpal tunnel release (UGCTR) or endoscopic carpal tunnel release (ECTR) in routine clinical practice.

**Methods:**

This prospective multicenter observational study enrolled patients with carpal tunnel syndrome who were treated with UGCTR or ECTR by experienced surgeons. Outcomes included Boston Carpal Tunnel Questionnaire Symptom Severity Scale and Functional Status Scale, pain severity (0–10 scale), opioid use, health-related quality of life, overall satisfaction, wound satisfaction, and adverse events through 3 months. Propensity score matching was performed to balance patient characteristics between groups.

**Results:**

Among 372 matched patients (186 per group), UGCTR was more commonly performed under wide-awake local anesthesia with no tourniquet (85.5% vs 30.1%), had a shorter incision length (5 vs 12 mm), and required less frequent suture closure (11.0% vs 100%). In contrast, procedure time was shorter with ECTR (8 vs 17 minutes). At 3 months, both groups showed significant improvement, with minor differences statistically favoring UGCTR (Boston Carpal Tunnel Questionnaire Symptom Severity Scale, 0.13 points; Functional Status Scale, 0.19 points; pain severity, 0.6 points; health-related quality of life, 0.05 points). Opioid use was lower after UGCTR (10.3% vs 39.7%). Overall satisfaction (92.1% vs 83.6%) and wound satisfaction (93.9% vs 88.3%) were higher with UGCTR. No serious adverse events occurred; nonserious events were less frequent with UGCTR (0% vs 5.9%).

**Conclusions:**

UGCTR and ECTR are safe and effective treatments for carpal tunnel syndrome. UGCTR was more commonly performed under wide-awake local anesthesia with no tourniquet and was associated with reduced opioid use and higher overall/wound satisfaction, whereas ECTR was associated with a shorter procedure time.

**Type of study/level of evidence:**

Therapeutic II.

Carpal tunnel syndrome (CTS) is the most common peripheral compressive neuropathy and a leading cause of disability.^1^, ^2^, ^3^, ^4^ Carpal tunnel release (CTR) is the definitive treatment for patients who do not respond to nonsurgical management, with more than 90% experiencing clinical improvement.^3^, ^4^, ^5^ CTR can be performed using several techniques including open CTR (OCTR), endoscopic CTR (ECTR), and ultrasound-guided CTR (UGCTR). ECTR and UGCTR are characterized by smaller incision sizes with different incision locations which may be desired by some patients.^6^

ECTR uses one or two small portals that permit instrument passage for visualization and division of the transverse carpal ligament. ECTR is associated with notable improvements in function and pain with a low risk of complications.^7^^,^^8^ Further, ECTR provides equivalent symptom relief to OCTR while allowing earlier functional recovery.^9^ UGCTR is a technique performed under real-time ultrasound guidance using a device inserted via a small incision proximal to the wrist crease. Multiple studies have demonstrated that UGCTR is associated with improvement in CTS symptoms and low complication rates.^10^, ^11^, ^12^ Compared to OCTR, patients who undergo UGCTR experience better functional gains, similar symptom relief, and a faster return to normal activities.^13^^,^^14^

In a retrospective single-center study, Moscato et al^15^ reported higher patient satisfaction at 2 months with UGCTR performed using wide-awake local anesthesia no tourniquet (WALANT) compared with ECTR performed under regional anesthesia. To our knowledge, no other study has directly compared UGCTR and ECTR. This prospective multicenter study aimed to compare the safety and effectiveness of UGCTR and ECTR in patients with CTS managed in routine clinical practice.

## Materials and Methods

### Ethics

The study protocol was reviewed and approved by an independent institutional review board. The study was prospectively registered at ClinicalTrials.gov (NCT060711468) and written informed consent was obtained from all participants before enrollment. Study reporting followed the Strengthening the Reporting of Observational Studies in Epidemiology guidelines and the Strengthening the Reporting of Observational Studies in Epidemiology extension for propensity score analyses.^16^^,^^17^

### Study design

The MISSION registry is a prospective, multicenter observational study designed to collect real-world outcomes of patients undergoing UGCTR, ECTR, or OCTR in routine clinical practice in the United States. This report presents a propensity score-matched comparison of 3-month outcomes in patients treated with UGCTR or ECTR by experienced surgeons.

### Patients

Eligible participants were adults who failed nonsurgical management and met three diagnostic CTS criteria: (1) a clinical diagnosis of unilateral or bilateral idiopathic CTS; (2) a CTS-6 score ≥12 points in the treated hand(s); and (3) a median nerve cross-sectional area ≥10 mm^2^ at the proximal carpal tunnel in the treated hand(s) measured using diagnostic ultrasound in the UGCTR group, or confirmatory electrodiagnostic testing based on laboratory-specific criteria in the ECTR group.^18^^,^^19^ Simultaneous bilateral procedures were permitted if both hands satisfied all criteria; unilateral procedures were permitted if only one hand qualified. Because this was a postmarket registry, no additional inclusion or exclusion criteria were imposed to reflect real-world practice.

Patients treated by surgeons who had performed ≥100 prior procedures using the respective technique were included in the analysis. All 9 ECTR investigators participating in the registry met this criterion, with a median of 11 years of experience (range, 7–34 years) and 1,800 previous procedures (range, 837–9,000). Among the 22 UGCTR investigators in the registry, 5 met this threshold, with a median of 3 years of experience (range, 1–7 years) and 380 previous procedures (range, 100–1,000). No investigator performed both UGCTR and ECTR because each specialized in a single technique.

### Procedure

The clinical setting and anesthetic approach were determined based on patient and surgeon preference. UGCTR was performed through a single incision in the region of the proximal wrist crease. A device (UltraGuideCTR, Sonex Health) incorporating a cutting blade and integrated balloons to protect adjacent neurovascular structures was used to divide the transverse carpal ligament from distal to proximal under ultrasound guidance. ECTR was performed using a single-portal (95%) or dual-portal (5%) technique, with the specific device and approach selected based on surgeon preference and experience. Procedural variables included laterality, dominant hand involvement, anesthesia type, tourniquet use, incision length, suture closure, procedure time (measured from skin incision to wound closure), intraoperative pain severity (0–10 scale),^20^ and concomitant procedures on the index hand.

### Outcome assessment

Data were collected on electronic case report forms at baseline, daily for the first 7 days to report early recovery experience, and at 2 weeks, 1 month, and 3 months for full outcome assessments. In-office follow-up visits were scheduled at the discretion of the treating surgeon. Symptomatic and functional recovery were assessed using the Boston Carpal Tunnel Questionnaire Symptom Severity Scale (BCTQ-SSS) and Functional Status Scale (BCTQ-FSS).^21^ Pain severity was assessed using a 0–10 scale,^20^ and postoperative opioid use and duration were recorded. The EuroQoL 5-Dimension 5-Level (EQ-5D-5L) questionnaire was used to assess health-related quality of life.^22^ Satisfaction with the procedure and the post-procedure wound were measured using a 5-point Likert scale (1 = very dissatisfied; 5 = very satisfied), with scores of 4 or 5 indicating satisfaction. Wound symptoms were assessed with patients reporting postprocedural wound sensitivity and pain. Adverse events were adjudicated by an independent medical reviewer and classified by seriousness according to Food and Drug Administration guidelines.^23^ An independent Data Safety and Monitoring Board provided study oversight to ensure participant safety.

### Propensity score matching methods

Because this was a nonrandomized study, propensity score methods were used to balance patient characteristics between groups. An independent data safety monitoring board prespecified 13 variables considered to have a potential impact on outcomes including age, sex, BCTQ-SSS score, CTS-6 score, thumb carpometacarpal (CMC) arthritis, inflammatory disease/arthritis, workers’ compensation status, current opioid use, chronic pain syndrome, depression/anxiety, prior CTR, diabetes mellitus, and CTR laterality (unilateral vs bilateral). Three variables were excluded from the final model: workers’ compensation status and prior CTR because of quasi-complete separation (ie, no events in one group) preventing model convergence, and thumb CMC arthritis because of nonrandom missing data in 28% of UGCTR patients related to its late addition to the case report forms.

Propensity scores were estimated using multiple logistic regression where treatment group served as the dependent variable. Matching (1:1 ratio) was performed using a greedy nearest neighbor algorithm without replacement and a caliper width of 0.20 SD of the logit of the propensity score. Covariate balance was assessed with the absolute standardized mean difference (SMD) before and after matching, calculated as the difference in means or proportions between groups divided by the pooled SD. An SMD < 0.25 was prespecified to indicate covariate balance between groups. Model specification and fitting procedures were performed before analysis of outcomes and followed established methods.^24^

### Statistical analysis

Power analysis indicated that 140 matched pairs would provide 90% power to detect a small-to-moderate effect size (Cohen’s *d* = 0.4) between groups at a two-sided alpha of 0.05. Allowing for up to 20% unmatched patients in the ECTR cohort, we planned to enroll at least 175 patients per group. Matched groups were analyzed using standard group-level methods because matching was performed to achieve covariate balance rather than to create 1:1 fixed pairs.^24^ Baseline characteristics were compared using chi-square tests for categorical variables and independent samples *t* tests for continuous variables. Longitudinal patient-reported outcomes were analyzed with linear mixed models that accounted for baseline values, repeated measures, and unilateral versus bilateral treatment, with results reported as least-squares means and 95% confidence intervals (95% CIs). Statistical testing for outcomes evaluated at multiple time points was performed at 3 months only to avoid multiple comparisons.

A double-robust sensitivity analysis was performed for BCTQ-SSS, BCTQ-FSS, pain severity, EQ-5D-5L, and overall satisfaction at 3 months. This analysis adjusted for baseline characteristics excluded from the prespecified propensity score model but that statistically differed between groups.^25^ These covariates were incorporated into the models and the results were compared with the primary analyses. A second sensitivity analysis excluded patients who underwent a concomitant procedure. A third sensitivity analysis compared adverse event rates between groups among all patients prior to propensity score matching to provide complete reporting of safety outcomes. Statistical significance was defined as a two-sided *P* < .05.

## Results

### Study population

**Figure 1:**
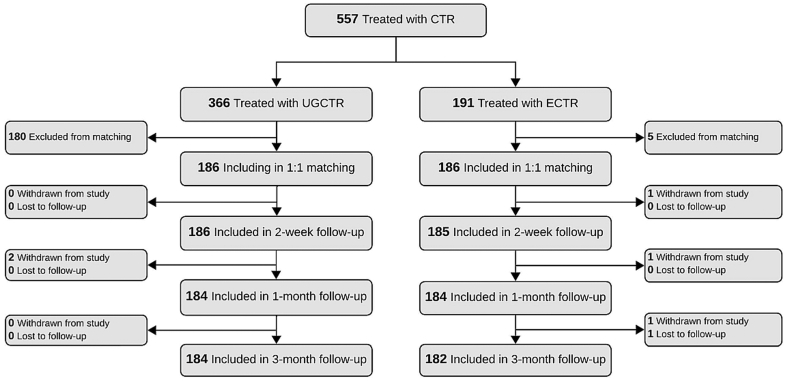
Study flow diagram. CTR, carpal tunnel release; ECTR, endoscopic carpal tunnel release; UGCTR, ultrasound-guided carpal tunnel release.

A total of 557 patients were treated between April 2024 and May 2025, including 366 with UGCTR at 5 sites and 191 with ECTR at 6 sites. After propensity score matching, 186 patients were included in each group. Follow-up compliance was high, with 98% of participants completing the 3-month assessment (Fig. 1).

### Patient characteristics

Propensity score matching achieved balance between treatment groups, with absolute SMDs < 0.25 and *P* values > .05 for all variables in the final model (Table 1). In the matched sample, most baseline characteristics were similar between groups (Table 2). The mean patient age was 57 years in both groups, and approximately two-thirds of patients were women. Almost half of the patients reported CTS symptoms lasting at least 2 years before treatment, most (58.9%) had bilateral disease, and the mean CTS-6 score was 19 in each group. Symptom severity, functional status, pain severity, and health-related quality of life were comparable between groups. Several baseline differences remained statistically significant when comparing UGCTR and ECTR, including the prevalence of degenerative arthritis (14.5% vs 7.0%; *P* = .02), trigger finger/thumb (9.7% vs 21.0%; *P* = .003), peripheral neuropathy (4.3% vs 11.3%; *P* = .01), work activity (61.7% vs 43.7% desk based work; *P* = .03), and previous CTR on the index hand (0% vs 2.2%; *P* = .04).

**Table 1:** Patient Characteristics Included in Propensity Score Modeling Before and After Matching∗

Characteristic	Unmatched Sample				Matched Sample			
	UGCTR (N = 366)	ECTR (N = 191)	SMD	*P* Value	UGCTR (N = 186)	ECTR (N = 186)	SMD	*P* Value
Variables Included in Model								
Age (y)	57.2 (13.1)	57.6 (14.3)	0.03	.76	56.7 (13.8)	57.4 (14.3)	0.05	.63
Female	65.0% (238/366)	68.1% (130/191)	0.06	.47	67.7% (126/186)	67.2% (125/186)	0.01	.91
BCTQ-SSS	3.12 (0.69)	3.18 (0.75)	0.08	.39	3.08 (0.71)	3.18 (0.76)	0.14	.18
CTS-6 score	19.9 (3.6)	18.7 (4.0)	0.30	.001	19.0 (3.6)	18.9 (4.0)	0.03	.74
Inflammatory disease/arthritis	6.3% (23/366)	6.3% (12/191)	0.00	>.99	8.1% (15/186)	6.5% (12/186)	0.06	.55
Current opioid use	6.6% (24/366)	3.7% (7/191)	0.13	.16	7.5% (14/186)	3.8% (7/186)	0.16	.12
Chronic pain disorder	2.7% (9/330)	6.3% (12/191)	0.17	.047	2.4% (4/169)	6.5% (12/186)	0.20	.06
Depression/anxiety	38.8% (142/366)	38.2% (73/191)	0.01	.89	41.4% (77/186)	38.2% (71/186)	0.07	.53
Diabetes mellitus	15.0% (55/366)	14.7% (28/191)	0.01	.91	14.0% (26/186)	14.5% (27/186)	0.02	.88
Bilateral treatment	12.3% (45/366)	20.4% (39/191)	0.22	.01	17.2% (32/186)	18.8% (35/186)	0.04	.69
Variables Excluded from Model								
Thumb CMC arthritis†	11.8% (31/262)	10.5% (20/191)	0.04	.65	12.6% (17/135)	10.2% (19/186)	0.07	.51
Workers’ compensation‡	0.0% (0/366)	1.6% (3/191)	0.18	.02	0.0% (0/186)	1.6% (3/186)	0.18	.08
Previous CTR (index hand)‡	0.0% (0/366)	2.1% (4/191)	0.21	.005	0.0% (0/186)	2.2% (4/186)	0.21	.04

BCTQ-SSS, Boston Carpal Tunnel Questionnaire Symptom Severity Scale; CMC, carpometacarpal; CTR, carpal tunnel release; CTS-6, Carpal Tunnel Syndrome-6; ECTR, endoscopic carpal tunnel release; SMD, standardized mean difference; UGCTR, ultrasound-guided carpal tunnel release.

∗ Values are expressed as mean±standard deviation or percentage (n/N).

† Variable was prespecified for inclusion in the propensity score model but was excluded because it was missing not at random in 28% of UGCTR patients because of its addition to the case report forms after study initiation. Data in the matched sample are provided for descriptive purposes.

‡ Variable was prespecified for inclusion in the propensity score model but was excluded because of quasiseparation (ie, zero events in one group), which prevented model convergence. Data in the matched sample are provided for descriptive purposes.

**Table 2:** Baseline Patient Characteristics∗

Characteristic	UGCTR (N = 186)	ECTR (N = 186)	*P* Value
Demographics			
Age (y)	56.7 (13.8)	57.4 (14.3)	.63
Female	67.7% (126/186)	67.2% (125/186)	.91
Race			.09
White	91.4% (169/185)	91.2% (165/181)	
Black	7.0% (13/185)	3.9% (7/181)	
Other race	1.6% (3/185)	5.0% (9/181)	
Body mass index (kg/m^2^)	31.2 (7.0)	30.7 (6.6)	.46
Medical history			
Depression/anxiety	41.4% (77/186)	38.2% (71/186)	.53
Previous corticosteroid injection (index hand)	28.5% (53/186)	20.4% (38/186)	.07
Degenerative arthritis (index arm/hand)	14.5% (27/186)	7.0% (13/186)	.02
Diabetes mellitus	14.0% (26/186)	14.5% (27/186)	.88
Thumb CMC arthritis (index hand)	12.6% (17/135)	10.2% (19/186)	.51
Trigger finger/thumb (index hand)	9.7% (18/186)	21.0% (39/186)	.003
Inflammatory disease/arthritis	8.1% (15/186)	6.5% (12/186)	.55
Current opioid use	7.5% (14/186)	3.8% (7/186)	.12
Cubital tunnel syndrome (index hand)	5.4% (10/186)	9.1% (17/186)	.16
Peripheral neuropathy	4.3% (8/186)	11.3% (21/186)	.01
Gout	3.2% (6/186)	6.5% (12/186)	.15
Cervical radiculopathy (index arm/hand)	2.7% (5/185)	1.6% (3/184)	.48
Chronic pain disorder	2.4% (4/169)	6.5% (12/186)	.06
Employment history			
Employed	61.8% (115/186)	55.4% (103/186)	.21
Work activity			.03
Desk based	61.7% (71/115)	43.7% (45/103)	
Repetitive light manual	20.9% (24/115)	28.2% (29/103)	
Heavy manual	17.4% (20/115)	28.2% (29/103)	
Workers compensation	0.0% (0/186)	1.6% (3/186)	.08
CTS history			
Previous CTR (index hand)	0.0% (0/186)	2.2% (4/186)	.04
Symptom duration			.89
≤3 mo	6.5% (12/185)	2.7% (5/186)	
>3 mo – 6 mo	6.5% (12/185)	8.1% (15/186)	
>6 mo – 2 y	37.8% (70/185)	40.9% (76/186)	
>2 y	49.2% (91/185)	48.4% (90/186)	
Bilateral carpal tunnel syndrome	54.8% (102/186)	62.9% (117/186)	.11
CTS-6 total score	19.0 (3.6)	18.9 (4.0)	.74
Patient-reported outcomes			
BCTQ-SSS (1–5 scale)	3.06 (0.70)	3.16 (0.76)	.18
BCTQ-FSS (1–5 scale)	2.43 (0.74)	2.46 (0.82)	.70
Pain severity (0–10 scale)	4.7 (2.7)	4.5 (2.7)	.46
EQ-5D-5L (0–1 scale)	0.72 (0.21)	0.68 (0.26)	.19

BCTQ-FSS, Boston Carpal Tunnel Questionnaire Functional Status Scale; BCTQ-SSS, Boston Carpal Tunnel Questionnaire Symptom Severity Scale; CMC, carpometacarpal; CTR, carpal tunnel release; CTS-6, Carpal Tunnel Syndrome-6; ECTR, endoscopic carpal tunnel release; EQ-5D-5L, EuroQoL 5-Dimension 5-Level; UGCTR, ultrasound-guided carpal tunnel release.

∗ Values are expressed as mean (standard deviation) or percentage (n/N).

### Procedure characteristics and analgesic use

Procedural details are presented in the Table 3. A total of 218 hands were treated with UGCTR, and 221 hands were treated with ECTR. Anesthetic management significantly differed between groups (*P* < .001). UGCTR was performed almost exclusively with WALANT (85.5%), whereas regional (10.8%) and general anesthesia (2.7%) were used infrequently. In comparison, ECTR was less frequently performed under local anesthesia (57.5%), divided between WALANT (30.1%) and non-WALANT local anesthesia (27.4%), and more frequently performed under general anesthesia (25.3%) or monitored anesthesia care (17.2%). UGCTR was associated with a smaller incision (5 vs 12 mm; *P* < .001) and sutures in only 11.0% of procedures compared with 100% of ECTR procedures (*P* < .001). Procedure duration was shorter in the ECTR group (8 vs 17 minutes; *P* < .001). Concomitant procedures were more common in the ECTR group (20.4% vs 8.6%; *P* = .001). Among patients treated with ECTR, concomitant procedure rates were comparable between cases performed using local anesthesia only and those performed using regional anesthesia, monitored anesthesia care, or general anesthesia (*P* = .96). Procedural pain was minimal in both groups. Patients in the ECTR group were prescribed opioids more frequently than those in the UGCTR group (60.3% vs 33.5%; *P* < .001). Postoperative opioid use was lower after UGCTR among all patients (10.3% vs 39.7%; *P* < .001) as well as among those who received an opioid prescription (30.6% vs 65.8%; *P* < .001), with a median 1 day of use in each treatment group. These difference remained statistically significant when patients who underwent a concomitant procedure were excluded from the analysis, with lower opioid use after UGCTR among all patients (6.5% vs 32.9%; *P* < .001) and among those who received an opioid prescription (21.6% vs 60.0%; *P* < .001).

**Table 3:** Procedural Characteristics∗

Characteristic	UGCTR (N = 186)	ECTR (N = 186)	*P* Value
Number of treated hands	218	221	
Bilateral CTR	17.2% (32/186)	18.8% (35/186)	.69
Dominant hand treated	62.9% (117/186)	64.5% (120/186)	.75
Anesthesia type†			<.001
Local	85.5% (159/186)	57.5% (107/186)	
Monitored anesthesia care	1.1% (2/186)	17.2% (32/186)	
Regional	10.8% (20/186)	0.0% (0/186)	
General	2.7% (5/186)	25.3% (47/186)	
Tourniquet use‡	0.9% (2/218)	69.2% (153/221)	<.001
WALANT	85.5% (159/186)	30.1% (56/186)	<.001
Incision length (mm)‡	5.3 (1.4)	12.1 (3.7)	<.001
Suture closure‡	11.0% (24/218)	100% (221/221)	<.001
Procedure time (minutes)	17 (13)	8 (7)	<.001
Procedural pain severity (0–10 scale)§	1.4 (2.1)	1.1 (2.0)	.21
Concomitant procedures (index hand)	8.6% (16/186)	20.4% (38/186)	.001
Trigger finger/thumb release	10	22	
Cubital tunnel release	6	6	
Injection	0	5	
CMC arthroplasty	1	3	
Cyst removal	1	2	
De Quervain tendon release	0	3	
Synovectomy	0	2	
Other procedures	0	3	

CMC, carpometacarpal; CTR, carpal tunnel release; ECTR, endoscopic carpal tunnel release; UGCTR, ultrasound-guided carpal tunnel release; WALANT, wide-awake local anesthesia no tourniquet.

∗ Values are expressed as mean (standard deviation) or percentage (n/N).

† Reported as the highest degree of anesthesia for combination anesthetic regimens.

‡ Reported on a per-hand basis.

§ Reported on a per-hand basis for patients who received local anesthesia only.

### Patient-reported outcomes at 3 months

**Figure 2:**
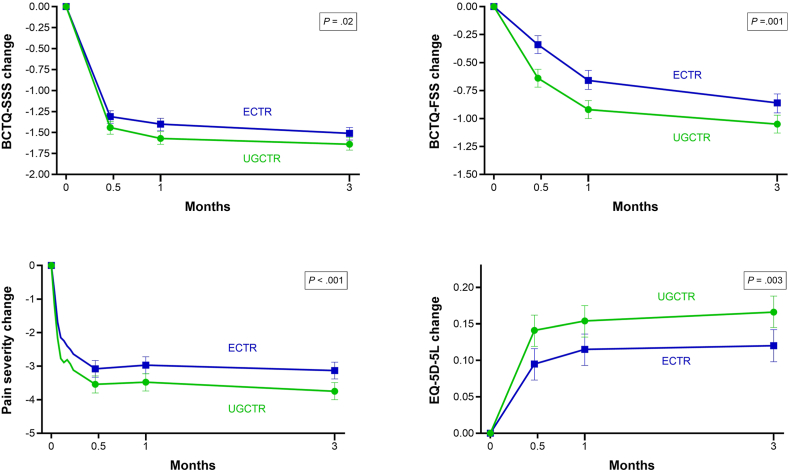
Changes in BCTQ-SSS, BCTQ-FSS, pain severity, and EQ-5D-5L over 3 months after UGCTR and ECTR. The mean change with 95% confidence interval over 3 months was statistically significant (P < .001) compared to baseline for all variables in both groups. Between-group P values for the 3-month comparison are provided within each panel. BCTQ-FSS, Boston Carpal Tunnel Questionnaire Functional Status Scale; BCTQ-SSS, Boston Carpal Tunnel Questionnaire Symptom Severity Scale; ECTR, endoscopic carpal tunnel release; EQ-5D-5L, EuroQoL 5-Dimension 5-Level.

At 3 months, BCTQ-SSS, BCTQ-FSS, pain severity, and EQ-5D-5L scores favored UGCTR, although the differences were minor (Fig. 2). The mean decrease in the BCTQ-SSS was 1.64 points with UGCTR and 1.51 points with ECTR (difference, 0.13; 95% CI, 0.02–0.23; *P* = .02). The mean decrease in the BCTQ-FSS was 1.05 points with UGCTR and 0.86 points with ECTR (difference, 0.19; 95% CI, 0.07–0.30; *P* = .001). Pain severity decreased by 3.7 points with UGCTR and 3.1 points with ECTR (difference, 0.6; 95% CI, 0.3–1.0; *P* < .001). EQ-5D-5L scores increased by 0.17 points with UGCTR and 0.12 points with ECTR (difference, 0.05; 95% CI, 0.02–0.08; *P* = .003).

**Figure 3:**
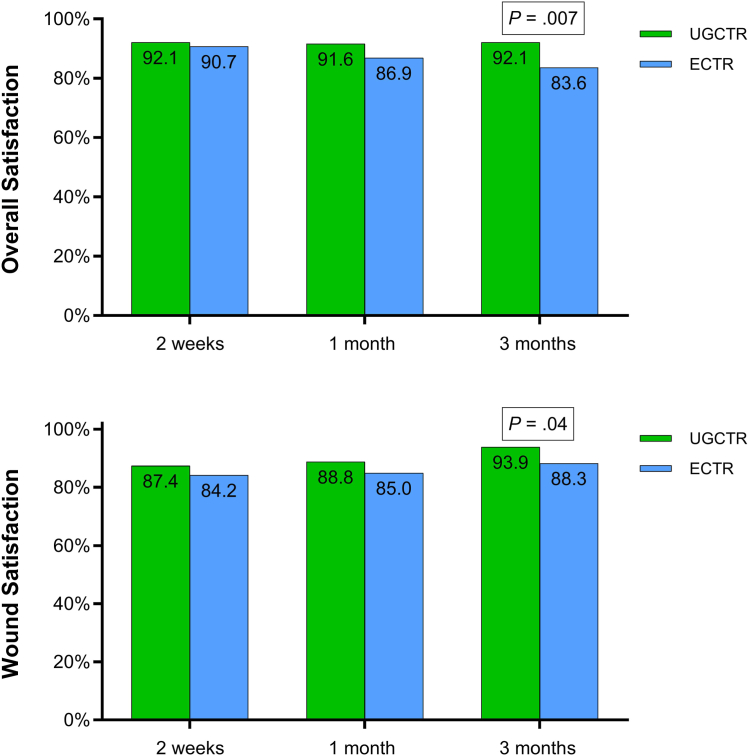
Overall satisfaction and wound satisfaction over 3 months after UGCTR and ECTR. Satisfaction defined as a score of 4 (satisfied) or 5 (very satisfied) on a 1–5 Likert scale. Between-group *P* values for the 3-month comparison are provided within each panel. ECTR, endoscopic carpal tunnel release; UGCTR, ultrasound-guided CTR.

Overall satisfaction (92.1% vs. 83.6%; *P* = .007) and wound satisfaction (93.9% vs. 88.3%; *P* = .04) at 3 months were higher in the UGCTR group (Fig. 3). Wound symptoms at 3 months were less frequent and severe with UGCTR (*P* < .001), with 61.2% versus 44.6% reporting no symptoms, 33.6% versus 43.7% reporting sensitivity, and 5.1% versus 11.7% reporting pain (Table 4).

**Table 4:** Postprocedural Wound Symptoms over 3 Months after UGCTR and ECTR

Follow-up	Symptoms	UGCTR	ECTR
1 wk	No symptoms	15.7%	12.7%
	Sensitivity	60.8%	48.8%
	Pain	23.5%	38.5%
2 wks	No symptoms	25.3%	22.8%
	Sensitivity	62.1%	59.1%
	Pain	12.6%	18.1%
1 mo	No symptoms	36.9%	23.4%
	Sensitivity	54.5%	56.1%
	Pain	8.6%	20.6%
3 mo∗	No symptoms	61.2%	44.6%
	Sensitivity	33.6%	43.7%
	Pain	5.1%	11.7%

ECTR, endoscopic carpal tunnel release; UGCTR, ultrasound-guided carpal tunnel release.

∗ *P* <001 between groups.

The sensitivity analysis that adjusted for baseline group differences in work activity, and the prevalence of degenerative arthritis, trigger finger/thumb, and peripheral neuropathy did not change the conclusions of the primary results for BCTQ-SSS, BCTQ-FSS, pain severity, EQ-5D-5L, and overall satisfaction at 3 months. In a sensitivity analysis that excluded patients who underwent a concomitant procedure, the primary conclusions also remained unchanged (Appendix S1, available online on the Journal’s website at https://www.jhsgo.org).

### Adverse events

No serious adverse events occurred in either group during the 3-month follow-up period. Nonserious adverse events were less frequent with UGCTR (0% vs 5.9%; *P* < .001). Adverse events in the ECTR group included cutaneous reactions (5), pain and/or numbness (3), and single cases of pisotriquetal instability, hand stiffness, and an incomplete release treated with revision surgery. In a sensitivity analysis including all 557 patients in the unmatched sample, no serious adverse events were reported in either group, and nonserious adverse events remained less frequent with UGCTR (0.5% vs 5.7%; *P* < .001).

## Discussion

This study demonstrated that both UGCTR and ECTR provided substantial symptom relief, functional improvement, and improved quality of life in patients with CTS over 3 months of follow-up. UGCTR was more commonly performed under WALANT and was associated with reduced postoperative opioid use and higher overall and wound satisfaction. Although improvements in BCTQ-SSS, BCTQ-FSS, pain severity, and EQ-5D-5L scores statistically favored UGCTR, the absolute differences between groups were modest. The primary benefit of ECTR was its shorter procedure duration. No serious adverse events occurred in either group. Collectively, these findings support the safety and effectiveness of both UGCTR and ECTR for the treatment of CTS.

The main differences between UGCTR and ECTR were primarily related to periprocedural factors and immediate recovery. UGCTR was usually performed with WALANT, and through a small incision that rarely required sutures. ECTR was associated with a shorter procedure time, but more often performed using a tourniquet, under general or monitored anesthesia care, through a larger incision requiring sutures, and with higher post-procedure opioid use. Beyond these periprocedural differences, patients treated with UGCTR reported higher overall satisfaction and wound satisfaction with fewer wound-related symptoms at 3 months. Although both groups showed large improvements in symptoms, function, and quality of life, the statistical differences between groups were of minor clinical importance. Together, these results suggest that the main distinctions between the techniques are procedure time, opioid use, and overall/wound satisfaction, rather than long-term symptomatic or functional outcomes.

Although studies comparing UGCTR and ECTR are limited, several indirect comparisons of these techniques have been reported.^15^ Ekhtiari et al^26^ used a decision tree model to demonstrate that UGCTR was associated with minor improvements in quality-adjusted life years compared to ECTR, whereas ECTR was associated with minor improvements compared to OCTR. All techniques were associated with successful outcomes and a low risk of complications. In a network meta-analysis of randomized trials evaluating seven CTR techniques, Elrosasy et al^27^ reported that, compared to OCTR, ECTR demonstrated superior functional outcomes and pain reduction, whereas UGCTR was associated with higher patient satisfaction. The current study extends these indirect findings by demonstrating that both UGCTR and ECTR provide effective treatment with distinct periprocedural characteristics rather than clear clinical superiority of either approach.

Strengths of this study include the prospective multicenter design, propensity score matching that controlled for preprocedural group differences, multiple sensitivity analyses that confirmed the primary findings, restriction of the analysis to patients treated by experienced surgeons, and use of validated patient-reported outcome measures. This study had several limitations that warrant further discussion. First, although propensity score matching accounted for differences in patient characteristics, unmeasured factors such as patient preferences and expectations may have influenced outcomes.^28^ Second, all UGCTR and ECTR procedures were performed by experienced surgeons, which may limit generalizability to those with less experience. However, the results in each group were consistent with prior reports that included physicians with varying levels of experience.^7^^,^^13^ Third, the choice of anesthesia and postoperative opioid prescribing patterns were determined by surgeon preference. Therefore, differences between groups in anesthesia use may be due to practice variation, patient-specific factors, or CTR technique. However, among patients who received an opioid prescription, opioid use remained lower after UGCTR than after ECTR. Thus, the difference in opioid use may relate to CTR technique differences, as well as patient recovery expectations, or other unmeasured factors. Fourth, although the favorable 3-month results are consistent with the period during which most clinical benefits of CTR are realized,^29^ continued follow-up will determine the long-term comparative effectiveness of the two techniques. Patients will be followed in the study for 2 years, and final results will be reported at that time. Finally, no investigator performed both UGCTR and ECTR procedures. Consequently, the choice of CTR technique was influenced by provider preference rather than patient-specific factors. However, the restriction to patients treated by surgeons with substantial prior experience in their respective technique may have limited the influence of operator proficiency on the comparative outcomes.

## Conflicts of Interest

Drs Miller, Watt, Niedermeier, and Marwin all have consultancy agreements with Sonex Health. No benefits in any form have been received or will be received by the other authors related directly to this article.
