# Supplementary material for: Prospective Multicenter Propensity Score-matched Comparison of Ultrasound-guided Versus Endoscopic Carpal Tunnel Release
Source: J Hand Surg Glob Online. 2026 Feb 27;8(3):100974. doi: 10.1016/j.jhsg.2026.100974 (PMC12966659; doi:10.1016/j.jhsg.2026.100974)
Supplement: Supplementary Material [file mmc1.docx]

**SUPPLEMENT**

**Double-robust Sensitivity Analysis**

**Methods**

A total of 372 matched participants (186 UGCTR; 186 ECTR) were included in the propensity score-matched cohort. Analyses were conducted according to the pre-specified primary models and repeated using double-robust models to assess the influence of baseline variables that statistically differed between groups after matching.

Five baseline variables statistically differed between the treatment groups after propensity score matching.

- - Employment (Unemployed, desk-based work, light manual work, heavy manual work)
  - Degenerative arthritis of upper limb on index hand (yes/no)
  - Peripheral neuropathy on index hand (yes/no)
  - Trigger finger on index hand (yes/no)
  - Previous CTR on the index hand

Continuous outcomes (BCTQ-SSS, BCTQ-FSS, pain severity, and EQ-5D-5L) were assessed as changes from baseline over 3 months, and overall satisfaction was assessed at 3 months, with additional adjustment for baseline propensity scores. Previous CTR on the index hand was excluded from the model because of quasi-complete separation (i.e., zero events in one group), which prevented model convergence. Treatment group differences in the primary analyses and double-robust sensitivity analyses were reported.

**Results**

Primary Analyses

BCTQ-SSS, BCTQ-FSS, pain severity, EQ-5D-5L, and overall satisfaction at 3 months demonstrated statistically significant differences in favor of UGCTR.

Double-robust Sensitivity Analyses

All outcomes statistically favored UGCTR in the double-robust sensitivity analysis (**Supplement Table 1**).

**Conclusions**

Baseline variables that remained statistically different between treatment groups after propensity score matching did not influence the primary conclusions for BCTQ-SSS, BCTQ-FSS, pain severity, EQ-5D-5L, or overall satisfaction at 3 months.

**Sensitivity Analysis Excluding Patients who Underwent a Concomitant Procedure**

**Methods**

A total of 372 matched participants (186 UGCTR; 186 ECTR) were included in the propensity score-matched cohort. Concomitant procedures were performed in 8.6% (16/186) of patients in the UGCTR group and 20.4% (38/186) of patients in the ECTR group. After excluding patients who underwent a concomitant procedure, outcomes of the remaining 170 patients in the UGCTR group were compared to the 148 patients in the ECTR group. Analyses were conducted according to the pre-specified primary models and repeated after excluding patients who underwent a concomitant procedure. Continuous outcomes (BCTQ-SSS, BCTQ-FSS, pain severity, and EQ-5D-5L) were assessed as changes from baseline over 3 months, and overall satisfaction was assessed at 3 months. Treatment group differences in the primary analyses and the sensitivity analyses excluding patients who underwent a concomitant procedure were reported.

**Results**

Primary Analyses

BCTQ-SSS, BCTQ-FSS, pain severity, EQ-5D-5L, and overall satisfaction at 3 months demonstrated statistically significant differences in favor of UGCTR.

Double-robust Sensitivity Analyses

All outcomes statistically favored UGCTR in the sensitivity analysis excluding patients who underwent a concomitant procedure (**Supplement Table 1**).

**Conclusions**

Exclusion of patients who underwent a concomitant procedure did not influence the primary conclusions for BCTQ-SSS, BCTQ-FSS, pain severity, EQ-5D-5L, or overall satisfaction at 3 months.

**Supplement Table 1**

Comparison of 3-Month Outcomes Between UGCTR and ECTR in the Primary Analyses and Sensitivity Analyses

| **Characteristic** | **Statistic** | **Primary**  **Analysis** | | **Double-robust**  **Sensitivity Analysis** | | **Concomitant Procedures Excluded Sensitivity Analysis** | |
| --- | --- | --- | --- | --- | --- | --- | --- |
|  |  | **Estimate** | **95% CI †** | **Estimate** | **95% CI †** | **Estimate** | **95% CI †** |
| BCTQ-SSS change | Mean difference* | -0.13 | -0.23, -0.02 | -0.14 | -0.25, -0.03 | -0.18 | -0.29, -0.06 |
| BCTQ-FSS change | Mean difference* | -0.19 | -0.30, -0.07 | -0.21 | -0.33, -0.10 | -0.22 | -0.34, -0.10 |
| Pain severity change | Mean difference* | -0.6 | -1.0, -0.3 | -0.6 | -1.0, -0.2 | -0.7 | -1.1, -0.4 |
| EQ-5D-5L change | Mean difference** | 0.05 | 0.02, 0.08 | 0.05 | 0.02, 0.08 | 0.04 | 0.01, 0.08 |
| Overall satisfaction | Relative risk*** | 2.07 | 1.20, 3.58 | 2.30 | 1.30, 4.06 | 2.12 | 1.20, 3.76 |

*Values are expressed as the difference between groups in the mean change for the variable of interest over 3 months, where negative values indicate greater decreases (improvements) with UGCTR than ECTR.

**Values are expressed as the difference between groups in the mean change for EQ-5D-5L over 3 months, where positive values indicate greater increases (improvements) with UGCTR than ECTR.

***A relative risk >1 indicates higher likelihood of not being satisfied with ECTR than UGCTR, defined as a score of 1 (very dissatisfied), 2 (dissatisfied) or 3 (neither satisfied nor dissatisfied) on a 1-5 Likert scale.

†For mean differences, a 95% CI that excludes 0 indicates a statistically significant difference between groups. For relative risk, a 95% CI that excludes 1 indicates a statistically significant difference between groups. In each model, all 95% CIs met these criteria and statistically favored UGCTR.

**Abbreviations:** BCTQ-FSS=Boston Carpal Tunnel Questionnaire Functional Status Scale; BCTQ-SSS=Boston Carpal Tunnel Questionnaire Symptom Severity Scale; ECTR=endoscopic carpal tunnel release; EQ-5D-5L=EuroQoL 5-Dimension 5-Level; UGCTR=ultrasound-guided carpal tunnel release; 95% CI=95% confidence interval.
